# Kinetic Jeans instability in FOG framework

**DOI:** 10.1038/s41598-026-44639-6

**Published:** 2026-03-18

**Authors:** Mritunjoy Das, Ahmed Atteya, Pralay Kumar Karmakar

**Affiliations:** 1 Department of Physics, Tezpur University, Napam, Sonitpur, Tezpur, 784028 Assam India; 2 Department of Physics, Faculty of Science, Alexandria University, P.O. 21511, Alexandria, Egypt

**Keywords:** Jeans instability, Structure formation, Modified gravity, Fourth order gravity, Kinetic theory, Astronomy and planetary science, Physics

## Abstract

The key mechanism triggering the complex dynamics of astrophysical structure formation in extensive evolutionary clouds is indeed sourced in the non-local gravitational (Jeans) instability. Motivated by recent astrophysical observations, establishing the growth of large-scale structures in the Universe, we herein propose a kinetic theory-based Jeans instability formalism in the fourth order gravity (FOG) framework. The analysis is modelled with the help of the canonical Boltzmann transport equation coupled with the FOG-modified Poisson equation. Our main goal lies in the inclusion of a scale-dependent free length parameter $\left( L \right)$ sourced in the FOG theory and its effects on the instability. The *L*-parameter governs the extent of the higher-order gravitational corrections over the classical Newtonian picture. Applying a standard local normal mode analysis, we derive the FOG-modified generalized linear dispersion relation. The dispersion analysis enables us to derive the critical Jeans mass for various structure-forming complex astroclouds, including Bok globules, diffuse molecular clouds, and giant molecular clouds. Interestingly, the resulting FOG-modified Jeans mass displays a non-monotonic density-dependency at larger $L$-values. This new gravitational behaviour restricts small-scale collapse, while promoting massive fragmentation, thereby leading to a preferentially smoother formation of large-scale structures without the inclusion of dark energy and dark matter. Our analysis has potential implications for the emergence of stellar mass distributions through the Jeans instability mechanism, with applicability in diverse astrophysical environments, fairly supported with real astronomical observations.

**Supplementary Information:**

The online version contains supplementary material available at 10.1038/s41598-026-44639-6.

## Introduction

Gravitational (Jeans) instability remains one of the most fundamental processes of material accretion, governing the formation and evolution of diverse astrocosmic structures. This instability was originally proposed by Sir J.H. Jeans in 1902^1^. He effectively integrated the principles of hydrodynamics with the Newtonian gravity theory to investigate the formation of self-gravitating astrostructures. This instability arises due to the inability of the outward acting thermal pressure to effectively counterbalance the inward acting self-gravity. The Jeans analysis provides a criterion for the collapse of an overdense region embedded within a uniform medium, identifying critical length and mass scales for the collapse process to initiate. The classical Jeans criterion is derived under the Newtonian gravity assumption and an idealized fluid treatment. These assumptions are often inadequate in more realistic astrophysical settings, and thus, new frameworks must be introduced. Again, it is also known that the theory of General Relativity (GR) alone would not be a viable gravity theory without the inclusion of dark energy and dark matter to explain various phenomena at the astrophysical and cosmological scales. Thus, in this context, the ${\Lambda}$-Cold Dark Matter (${\Lambda}$CDM) model has emerged as the standard cosmological model. Here, the cold dark matter accounts for the unseen gravitational effects in the galaxies and clusters^2,3^. Again, the dark energy, modelled as the cosmological constant ${\Lambda}$, drives the observed late-time acceleration of the Universe^4^.

In recent years, various observational data have firmly established the existence of large-scale structures in the Universe. Major astrophysical missions such as the Planck mission^5^, Sloan Digital Sky Survey (SDSS)^6^, Dark Energy Survey (DES)^7^, eROSITA^8^, along with the ongoing Euclid mission, have provided high-precision data, revealing the large-scale distribution of matter^9,10^. These observations support the hierarchical nature of structure formation and highlight the need for a theoretical framework capable of describing the gravitational collapse of extended and diffuse matter distributions into large-scale astrostructures. The confirmation of such vast structures has renewed interest in the theoretical foundations of structure formation. As a consequence, the classical Jeans instability framework has been extended and revisited in various cosmological and astrophysical contexts. This includes studies involving dark matter background^11,12^, accelerated cosmic expansion^13^ and so forth.

While the ΛCDM model explains the growth of such structures through cold dark matter-driven gravitational clustering, it is fundamentally reliant on the existence of dark energy and dark matter. However, these components are yet to be detected experimentally^2^. This lack of experimental confirmation has led to the increasing interest in modified gravity theories as an alternative approach.

It is noteworthy that various modified models of gravity have been proposed to explain the diverse astrophysical measurements and phenomena in the recent past^14–28^. These alternative gravity frameworks have been implemented to study the various gravity-driven instabilities^29–41^. In fact, such investigations offer deeper elements for understanding structure formation processes, aligning with various astronomical and astrometric observations^42^. Moreover, recent JWST observations have revealed a population of unusually luminous and massive star-forming galaxies at very high redshifts, whose abundance exceeds expectations from standard models^43^. Thus, these observations motivate further theoretical modelling to investigate the physical processes governing the Jeans collapse and fragmentation in massive star-forming environments.

In continuation of the above research scenarios, the present work sheds light on the formation mechanism of such large-scale structures within a modified gravity setting. In this study, we employ the fourth order gravity (FOG) model for the first time to investigate the existence and formation of large-scale structures^18^. The FOG model formulation was originally formulated to account for the observed flatness of the galaxy rotation curves and to explain the presence of a universal acceleration without introducing the concept of dark energy^18^. The FOG theory introduces higher-order curvature corrections to the Einstein field equations^18^. Motivated by the incorporation of higher-order curvature effects sourced in spatial inhomogeneities, we propose a new model formalism to refine the traditional Newtonian gravity-based Jeans instability in the FOG framework incorporating the above-mentioned corrections. This formulation enables a systematic investigation on the impact of the non-Newtonian gravity refinements on the critical (threshold) conditions for triggering gravitational collapse in diverse astrophysical systems, leading to structure formation.

In the weak-field non-relativistic limit, the FOG theory introduces a biharmonic correction to the traditional Newtonian Poisson Eqs^18,44^. With this biharmonic correction, we derive the FOG-modified critical thresholds for the Jeans collapse. To further advance the theoretical modelling of the instability, we adopt a kinetic theory approach as opposed to the conventional fluid-based treatment. The hydrodynamic approach assumes collisional equilibrium, and the system is treated as a compressible fluid. On the contrary, the kinetic approach allows for a more fundamental characterization of the system dynamics by tracking the evolution of the particle distribution in phase space. This formalism is particularly well-suited for describing collisionless or weakly collisional systems, where the non-local gravitational interactions dominate over local collisional effects. Thus, in this study, we investigate the influence of the FOG corrections on the Jeans instability within a kinetic framework. We derive the FOG-modified critical limits for initiating the Jeans collapse process. A modal analysis is also performed to investigate the hierarchical fragmentation of the self-gravitational astroclouds. This research offers new insights into understanding the role of such modified gravity formalisms on the structure formation processes in our Universe. Beyond its theoretical interests, our proposed framework motivates investigation for a range of astrophysical settings. The scale-dependent gravitational instability thresholds introduced in this work may influence the mass distribution of various compact astroobjects. These modified critical limits may regulate the fragmentation scales in dense accretion disks, and potentially affect early formation pathways of massive black hole seeds.

## Kinetic model formalism

Here, we investigate the self-gravitational collapse of a collisionless system, and identify the instability thresholds within the FOG framework. Application of the Boltzmann-Vlasov equation coupled with the FOG-modified Poisson equation, we derive new critical Jeans limits and a FOG-modified dispersion relation. The collisionless Boltzmann-Vlasov equation governs the evolution of the matter distribution function $f\left( {\bar {r},\bar {v},t} \right)$, and is given in the following form as

1 $${\partial _t}f\left( {\bar {r},\bar {v},t} \right)+\bar {v}.\nabla f\left( {\bar {r},\bar {v},t} \right) - \nabla \psi .{\partial _{\bar {v}}}f\left( {\bar {r},\bar {v},t} \right)=0.$$

The absence of a collisional term implies that the local particle collisions are negligible compared to the collective effects of the self-gravitational force. This assumption is justified as the dominant mechanism in the Jeans instability is the interplay between the self-gravitational attraction and outward acting internal thermal pressure. Equation (1) does not rely on collisions but instead focuses on the collective dynamics of the particles under the influence of gravity.

Furthermore, in gravitationally bound stellar systems, collisions are very rare, and the gravitational interactions dominate over local interactions. Subsequently, astrocloudic systems such as the giant molecular clouds (GMCs), Bok globules, and so forth are collisional at small scales, but these collisions can be neglected in comparison to the large-scale gravitational collapse. Thus, the kinetic description of Jeans instability can be applied to study both astrocloudic structures and stellar systems. The dynamics of the matter distribution function $f\left( {\bar {r},\bar {v},t} \right)$ in phase-space is influenced by the FOG-modified gravitational potential $\left( \psi \right)$. The FOG model is described by the following effective field equations^18^, given as

2 $${R_{{\mathrm{\boldsymbol{\upmu}\boldsymbol{\upnu}}}}} - \frac{1}{2}{g_{{\mathrm{\boldsymbol{\upmu}\boldsymbol{\upnu}}}}}R=\frac{{8{\mathrm{\boldsymbol{\uppi}}}G}}{{{c^4}}}{T_{{\mathrm{\boldsymbol{\upmu}\boldsymbol{\upnu}}}}}+{L^2}R_{{{\mathrm{\boldsymbol{\upmu}\boldsymbol{\upalpha}\boldsymbol{\upnu}\boldsymbol{\upbeta}}}}}^{{;{\mathrm{\boldsymbol{\upalpha}\boldsymbol{\upbeta}}}}}.$$

The additional term $\left( {{L^2}R_{{{\mathrm{\boldsymbol{\upmu}\boldsymbol{\upalpha}\boldsymbol{\upnu}\boldsymbol{\upbeta}}}}}^{{;{\mathrm{\boldsymbol{\upalpha}\boldsymbol{\upbeta}}}}}} \right)$ modifies the Einstein field equations by introducing a correction dependent on the Riemann tensor. This leads to fourth-order derivatives of the metric, distinguishing FOG theory from the standard GR description. The higher-order corrections introduced in the FOG theory arise as effective field equations, in which case there may not be an underlying action principle^44^. Notably, the term ${L^2}R_{{{\mathrm{\boldsymbol{\upmu}\boldsymbol{\upalpha}\boldsymbol{\upnu}\boldsymbol{\upbeta}}}}}^{{;{\mathrm{\boldsymbol{\upalpha}\boldsymbol{\upbeta}}}}}$ is not a boundary term and contributes directly to the gravitational dynamics leading to a modified Poisson equation in the weak-field limit^18^. Following the phenomenological formulations of the FOG theory, the *L*-parameter is introduced as an effective gravitational scale length or free length parameter, defined as $L\sim {\left( {GM/c{H_0}} \right)^{1/2}}$^18,45^. Here, $G=6.67 \times {10^{ - 11}}{\mathrm{~N~}}{{\mathrm{m}}^2}{\mathrm{~k}}{{\mathrm{g}}^{ - 2}}$ is the gravitational coupling constant, *c* being the vacuum speed of light, *M* is the system mass, and ${H_0}$ is the present value (also termed as the Hubble constant) of the Hubble parameter, $H\left( t \right)$. In our analysis, we consider the constant of proportionality to be unity and have set $L={\left( {GM/c{H_0}} \right)^{1/2}}$ throughout the study.

Furthermore, we emphasize that the *L*-parameter is treated as an external phenomenological parameter that remains fixed during linear perturbation analysis. It is worth noting that various constraints on the *L*-parameter have been proposed in the recent literature^45,46^. However, such constraints are not imposed in this study, as the primary aim of this work is to investigate the influence of the higher-derivative gravitational corrections, characterized by the *L*-parameter on triggering astrostructure formation in the weak-field regime. Now, to obtain the Newtonian weak-field gravity limit in this theory, we use the following metric

3 $$d{s^2}=\left( {1+2\frac{\psi }{{{c^2}}}} \right){c^2}d{t^2} - d{x^2} - d{y^2} - d{z^2}.$$

Here, the only non-zero component of the energy-momentum tensor is $T_{0}^{0}={c^2}\rho$, where $\rho$ denotes the matter density of the system. In this limit, Eq. (2) reduces to the following fourth-order biharmonic correction to the gravitational Poisson equation^18^, cast as

4 $${\nabla ^2}\psi - {L^2}{\nabla ^4}\psi =4\pi G\left( {\rho - {\rho _0}} \right).$$

In Eq. (4), the term, ${L^2}{\nabla ^4}\psi$, originates basically due to the higher-derivative (fourth-order) gravity effects. In a special case, if $L=0$, Eq. (4) reduces to the usual gravitational Poisson equation, ${\nabla ^2}\psi =4\pi G\rho$, which is based on the inverse-square Newtonian gravity for point-gravitating sources^18,44^. Here, $\rho =\smallint f\left( {\bar {r},\bar {v},t} \right){d^3}v=\smallint f\left( {\bar {r},\bar {v},t} \right)d\bar {v}$, where $d\bar {v} \equiv {d^3}v=d{v_x}~d{v_y}~d{v_z}$ denotes the volume element in velocity space^47,48^. The velocity integration is performed for $v \in \left( { - \infty ,+\infty } \right)$, in accordance with the Maxwell-Boltzmannian distribution.

It is worth noting that the biharmonic modification of the gravitational Poisson equation also arises in the framework of gravitational quadrupole polarization^49^. The term “gravitational polarization” refers to an applied concept employed to conceive how a gravitating medium reacts against the action of an external gravitational field (like electric polarization in dielectric materials). In that approach, macroscopic averaging of the Einstein field equations is performed for a gravitating medium exhibiting quadrupole polarization. In the weak-field limit, this treatment leads to a modified Poisson equation containing a fourth-order (biharmonic) correction to the Newtonian gravitational potential. Although the physical origin of the correction differs from the higher-curvature modification considered in the present FOG framework. Both approaches lead to the same structural biharmonic form of the gravitational Poisson equation.

In the above context, it may be an interesting point to note further that, for specific geometries, the effective radial gravity acceleration resulting from the FOG formalism in generic notations^18^ reads as

5 $${g_{FOG}}\left( r \right)= - \frac{{GM\left( r \right)}}{{{r^2}}}C\left( r \right).$$

The FOG correction factor against the Newtonian one is given in a usual form as

6 $$C\left( r \right)=1+\sqrt {\frac{{{M_0}}}{M}} \left[ {1 - {e^{\left( { - r/L} \right)}}\left( {1+r/L} \right)} \right],$$

where, ${M_0}$ is a constant signifying a system mass-scaling law^18^. For example, if one takes ${M_0}={10^{12}}{M_ \odot }$, ${M_ \odot }=$ solar mass; it is possible to fit the flat rotation curves of a large galactic class, even without invoking any kind of effects of dark matter^18^. Furthermore, it should also be noted that while phenomenological softening schemes, such as the Plummer potential (smoothened), regularize the Newtonian singularity by the introduction of a softening length (Plummer radius, ${r_0}$)^50^. The FOG framework suppresses short-scale gravitational responses through higher-derivative gravity corrections. It provides a natural stabilization mechanism for extended self-gravitating systems relevant to diverse structure formation processes.

In this work, we use the above-mentioned Poisson equation (Eq. (4)) for the model closure, thus allowing us to study the self-gravitational collapse of astrophysical systems and derive an FOG-modified dispersion relation. The primary goal is to study the dependency of the instability on the *L*-parameter. The relation acquired is used to analyse different modes of instability in the FOG framework.

In the proposed model formalism, we restrict ourselves to the weak-field, non-relativistic limit of the FOG theory, where the biharmonic Poisson equation emerges. Accordingly, the kinetic theory employed is Newtonian, and no fully relativistic kinetic formulation is attempted. This approximation is well justified, since the Jeans instability is fundamentally a low-velocity, non-relativistic phenomenon in which the characteristic dynamical timescales are much longer than the relativistic ones. The weak-field limit adopted in this work is consistent with the standard treatment of the structure formation process in both the classical Newtonian and modified gravitational frameworks across a wide range of astrocosmic scales.

In a hydrostatic homogeneous equilibrium configuration, one finds $\psi ={\psi _0}$, $\nabla {\psi _0}=0$, ${\nabla ^2}{\psi _0}=0$, and ${L^2}{\nabla ^4}{\psi _0}=0$. As a result, Eq. (4) yields $\rho ={\rho _0} \ne 0$, thereby modelling the Jeans swindle^51,52^. Here, $\psi ={\psi _0}=0$ denotes the equilibrium potential value and $\rho ={\rho _0} \ne 0$ is the equilibrium material density. In this context, it is worth mentioning that the “Jeans swindle” is an ad-hoc homogenization assumption, extensively useful to model inhomogeneous self-gravitating astrophysical media. It simplifies model formalisms by ignoring the mathematical complications arising from the zeroth-order gradient effects. This swindle implies that the gravitational potential distribution in self-gravitating media is not exclusively sourced by the local material density, but by its fluctuations only^51,52^. This assumption enables researchers to replace the complicated non-local inhomogeneous equilibrium density of self-gravitating systems with the corresponding local homogeneous one without any loss of generality. It implicates that the gravitational Poisson equation describes the mathematical relationship between the perturbed potential and the perturbed density fields only. The word “swindle” in “Jeans swindle” indeed refers to the fact that there is usually no formal physical justification in ignoring the unperturbed (zeroth-order) force field effects in treating such inhomogeneous media. The assumption of background homogeneity here actually originates from the considered local hydrostatic (field-free) equilibrium configuration. However, it has later been reported that there exists a formal justification for the Jeans swindle on the cosmological scales arising from the correct coupling between the mean matter density field and the expansion of the Universe^53^.

The applicability of the background homogeneity assumption (Jeans swindle) in the FOG framework here is not more restricted than the extensively studied Newtonian one. For a homogeneous background density, the additional higher-order (biharmonic) correction term does not introduce any new constraints on the equilibrium configuration. The fourth-order correction contributes only in the presence of spatial inhomogeneities. This new correction term in the gravitational Poisson equation only affects the density perturbations rather than the uniform background matter distribution. As a result, the assumption of background homogeneity remains well consistent in the FOG framework.

Now, the relevant physical parameters $\left( A \right)$ describing the system dynamics are made to undergo small-scale (linear) perturbations (${A_1}$) around their equilibrium values $\left( {{A_0}} \right)$. The perturbations are assumed to develop as homologous sinusoidal signals. Thus, the distribution function and the gravitational potential in our model system can be respectively expressed as

7 $$f\left( {\bar {r},\bar {v},t} \right)={f_0}+{f_1},$$

8 $$\psi \left( {\bar {r},t} \right)={\psi _0}+{\psi _1}.$$

Here, the planar wave perturbations are of the standard sinusoidal form ${A_1}\sim {A_{10}}{e^{i\left( {kx - \omega t} \right)}}$, with ${A_{10}}$ defining the amplitude of the perturbations. The linear wave analysis introduces a new wave-space $\left( {\omega ,k} \right)$, in which the differential operators are auto-transformed as ${\partial _t}~ \to - i\omega$ and $\nabla \to i\bar {k}$. Here, $\omega$ and *k* are the Jeans angular frequency and the Jeans wavenumber, respectively.

Now, substituting Eq. (7) and Eqs. (8) into  (1) and Eq. (4), respectively, and Fourier transforming the perturbation variables, the first-order system of equations can be recast in the Fourier space as

9 $$- i\omega {f_1}+\bar {v}.\left( {i\bar {k}{f_1}} \right) - \left( {i\bar {k}{\psi _1}} \right).{\partial _{\bar {v}}}{f_0}=0,$$

10 $$- {k^2}{\psi _1} - {L^2}{k^4}{\psi _1}=4\pi G\smallint {f_1}\left( {\bar {r},\bar {v},t} \right)d\bar {v},$$

Now, substituting the expression for ${f_1}$ from Eq. (9) in Eq. (10) and imposing $\overline {{\left| k \right|}} =\left( {k,0,0} \right)=\left( {{k_x},0,0} \right)$ in the velocity direction $\overline {{\left| v \right|}} =v={v_x}$, one obtains the generalized linear dispersion relation for the collective Jeans mode under consideration, cast in generic notations as

11 $$1+{L^2}{k^2}+\frac{{4\pi G}}{{{k^2}}}\smallint \left( {\frac{{k~}}{{kv - \omega }}{\partial _v}{f_0}} \right)dv=0.$$

The collapsing system is considered to be isothermal. This isothermal assumption can be used in the case of Jeans collapse due to the radiative cooling mechanism^54–57^. In many astrophysical environments, the system can effectively radiate away the heat generated during the collapse process. Moreover, the timescale of the Jeans collapse is very large when compared to the timescale of heat dissipation, allowing an isothermal treatment. Again, assuming a local thermodynamical equilibrium, the background particle distribution function can be described using the Maxwell-Boltzmann distribution given as

12 $${f_0}\left( {{v_x}=v} \right)=\frac{{{\rho _0}}}{{\sqrt {2\pi {\sigma ^2}} }}{e^{\left( { - {v^2}/2{\sigma ^2}} \right)}}.$$

Here, ${\rho _0}={m_p}{n_H}\mu$ denotes the equilibrium matter density, where ${m_p}$ is the proton mass, ${n_H}$ is the number of particles measured in ${{\mathrm{m}}^{ - 3}}$, and $\mu$ is the mean molecular weight. Again, $\sigma =\sqrt {{k_B}T/{m_H}}$ denotes the thermal dispersion velocity of the particles with ${k_B}=1.38 \times {10^{ - 23}}~{\mathrm{J}}{{\mathrm{K}}^{ - 1}}$ being the Boltzmann constant, and *T* denoting the temperature of the system.

Now, applying Eq. (12) in Eq. (11), we get

13 $$1+{L^2}{k^2} - \frac{{4\pi G}}{{{k^2}}}\frac{{{\rho _0}}}{{\sqrt {2\pi } {\sigma ^3}}}k\smallint \frac{{v{e^{\left( { - {v^2}/2{\sigma ^2}} \right)}}}}{{kv - \omega }}dv=0.$$

Here, putting $L=0$, Eq. (13) reduces to the Newtonian dispersion relation^47^. In the Newtonian case, we can infer the limit for the self-gravitational collapse by setting $\omega =0$, and computing the maximum wavelength of the perturbations supported by the system, above which the system collapses. Thus, we obtain the Jeans wavenumber in the Newtonian regime, and it is given as

14 $$k={k_J}=\frac{{\sqrt {4\pi G{\rho _0}} }}{\sigma }.$$

Now, the Jeans mass $\left( {{M_J}} \right)$ is defined by the mass enclosed in a sphere of diameter ${\lambda _J}=2\pi /{k_J}$, obtaining

15 $${M_J}=\frac{\pi }{6}\sqrt {\frac{1}{{{\rho _0}}}{{\left( {\frac{{\pi {\sigma ^2}}}{G}} \right)}^3}} .$$

Now, in the FOG formalism $\left( {L \ne 0} \right)$, putting $\omega =0$ in Eq. (13) we get a quadratic equation in terms of ${k^2}$, and is cast as

16 $${k^4}{L^2}{\sigma ^2}+{k^2}{\sigma ^2} - 4\pi G{\rho _0}=0.$$

Using the standard quadratic formula, we obtain two mathematical solutions for the FOG-modified Jeans wavenumber, given as

17 $$k=k_{{{J_ \pm }}}^{{FOG}}={\left( {\frac{{ - {\sigma ^2} \pm \sqrt {{\sigma ^4}+16{L^2}{\sigma ^2}\pi G{\rho _0}} }}{{2{L^2}{\sigma ^2}}}} \right)^{1/2}}.$$

Again, the two mathematically possible solutions for the FOG-modified Jeans critical masses $\left( {M_{{{J_ \pm }}}^{{FOG}}} \right)$ are defined by the mass enclosed by the spheres of diameter ${\mathrm{\boldsymbol{\uplambda}}}_{{{J_ \pm }}}^{{FOG}}=2\pi /k_{{{J_ \pm }}}^{{FOG}}$, and are respectively cast as

18 $$M_{{{J_ \pm }}}^{{FOG}}=\left( {\frac{{4{\rho _0}{\pi ^4}}}{3}} \right){\left( {\frac{{2{L^2}{\sigma ^2}}}{{ - {\sigma ^2} \pm \sqrt {{\sigma ^4}+16{L^2}{\sigma ^2}\pi G{\rho _0}} }}} \right)^{3/2}}.$$

In the standard Newtonian case of the Jeans instability analysis, there exists a single critical limit for unstable modes. In the FOG framework, the ${k^4}$-term in Eq. (16) formally yields two mathematical solutions $\left( {k_{{{J_ \pm }}}^{{FOG}}} \right)$. The appearance of multiple solutions is expected as higher-order gravitational theories allow additional degrees of freedom^30^. However, only the positive real root $\left( {k_{{{J_+}}}^{{FOG}}} \right)$ corresponds to a physically meaningful instability threshold. The second solution $\left( {k_{{{J_ - }}}^{{FOG}}} \right)$ is purely imaginary under real astrophysical parameters and has therefore no physical significance. We also note that the appearance of the ${k^4}$-term in the dispersion relation is not unique to the FOG case. It also arises in the quantum treatment of the Jeans instability, where the quantum pressure corrects the classical counterpart^50,58^. In the present case, however, the contribution from the ${k^4}$-term originates solely from the biharmonic modification to the Poisson equation. The supplementary information provided further elaborates that the generalized Jeans wavenumber in the FOG theory is uniquely given by $k_{{{J_+}}}^{{FOG}}$, which reduces to ${k_J}$ in the limit $L \to 0$. So, the associated critical mass $M_{{{J_+}}}^{{FOG}}$ can be signified as the generalization of ${M_J}$, and will be referred simply as $M_{J}^{{FOG}}$ in the subsequent sections for analytic simplicity. Thus, the newly derived FOG-modified Jeans critical mass limit in the usual symbolism is cast as

19 $$M_{{J+}}^{{FOG}}=M_{J}^{{FOG}}=\left( {\frac{{4{\rho _0}{\pi ^4}}}{3}} \right){\left( {\frac{{2{L^2}{\sigma ^2}}}{{ - {\sigma ^2}+\sqrt {{\sigma ^4}+16{L^2}{\sigma ^2}\pi G{\rho _0}} }}} \right)^{3/2}}.$$

## FOG-modified Jeans mass

To gain a deeper insight into the FOG-modified Jeans instability behaviour, it is essential to examine the modified critical limit $\left( {M_{J}^{{FOG}}} \right)$ derived in the previous section, defined by Eq. (19). Here, we investigate the effects of the model-relevant parametric variations on $M_{J}^{{FOG}}$ for typical structure-forming astrocloudic systems. To ensure a dimensionally-invariant form of graphical representation, we normalize the relevant parameters accordingly. $M_{J}^{{FOG}}$ is normalized with respect to the typical mass of GMCs $\left( {{M_{GMC}}={{10}^{34}}{\mathrm{~kg}}} \right)$^59^, and the background density is normalized with a characteristic GMC density $\left( {{\rho _{GMC}}={{10}^{ - 20}}{\mathrm{~kg}}/{{\mathrm{m}}^3}} \right)$^59,60^.

**Fig. 1:**
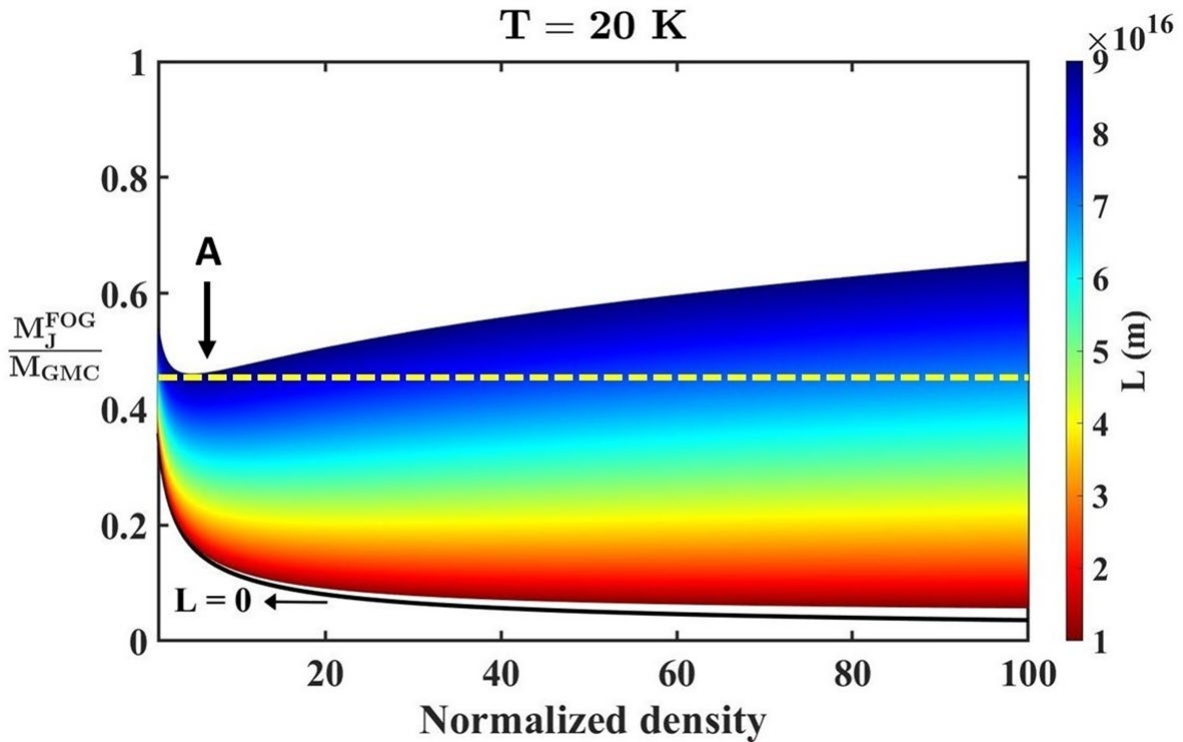
Profile of the normalized FOG-modified Jeans mass with variation in the normalized matter density for different indicated *L*-values with “*A*” denoting the minimum $M_{J}^{{FOG}}/{M_{GMC}}$ for $L=9 \times {10^{16}}$ m.

As shown in Fig. 1, we present the variation of the normalized FOG-modified critical Jeans mass $\left( {M_{J}^{{FOG}}/{M_{GMC}}} \right)$ as a function of the normalized background material density and the *L*-parameter for a fixed temperature $\left( {T=20~{\mathrm{K}}} \right)$. The choice of $T=20$ K in Fig. 1 is physically motivated by typical conditions in cold Giant Molecular Clouds (GMCs). The cloud temperature of GMCs typically varies as $T\sim \left( {10 - 20} \right)$ K^31,59,60^. Examples of molecular cloud environments with $T\sim 10$ K include clouds identified in the Galactic Ring Survey, such as GRSMC G 025.19-00.26 and GRSMC G 019.84-00.41^31^. Since the present analysis primarily aims at exploring the gravitational instability dynamics in molecular cloud environments, adopting $T=20$ K provides a representative and observationally consistent value within this regime. We emphasize that this choice does not represent a special or fine-tuned parameter, but rather a standard characteristic temperature of cold star-forming regions. The colour-gradient map represents different values of the *L*-parameter ranging from $1 \times {10^{16}}$ m to $9 \times {10^{16}}$ m (Fig. 1). The magnitude of the *L*-values is estimated for typical GMCs using the standard definition adopted in the FOG formalism^18^. Thus, the parameter is explored phenomenologically within astronomically motivated weak-field environments.

Besides, as depicted in Fig. 1, the black curve corresponds to the standard Newtonian prediction $\left( {L=0} \right)$. It is evident that, for $L>0$, the modified critical limit is greater than the Newtonian baseline (Fig. 1). We also see that, at lower background density, the critical mass limit attains the maximum value for the Newtonian gravity theory $\left( {L=0} \right)$. Furthermore, a particular notable deviation from the Newtonian scenarios emerges at higher *L*-values, where the critical limits predicted by the FOG theory do not follow the typical monotonic decrease with enhanced matter density, as expected in the Newtonian regime. In the FOG case, $M_{J}^{{FOG}}/{M_{GMC}}$ initially drops to a minimum value (indicated by a yellow dashed line and a directional arrow for $L=9 \times {10^{16}}$ m), and subsequently exhibits a gradual upward trend with increasing matter density. Here, “*A*” denotes the minimum $M_{J}^{{FOG}}/{M_{GMC}}$ for $L=9 \times {10^{16}}$ m. This non-monotonic behaviour suggests the existence of an intermediate density regime where the gravitational threshold for the collapse initiation is minimized. Physically, it indicates that, for a sufficiently large *L*-value (massive astrosystem), the gravitational response is scale-sensitive in such a way that the collapse becomes most efficient at moderate densities. This intriguing deviation from the Newtonian Jeans scenarios highlights the interplay between the background matter density fields and those by the FOG-induced corrections in the proposed model analysis. A detailed analytical investigation of the non-trivial density dependency of $M_{J}^{{FOG}}$ is presented in the supplementary information. It provides a comprehensive criticality analysis in both the low-density and high-density regimes, confirming the non-monotonic behaviour of $M_{J}^{{FOG}}$ with matter density as already clearly illustrated in Fig. 1.

**Fig. 2:**
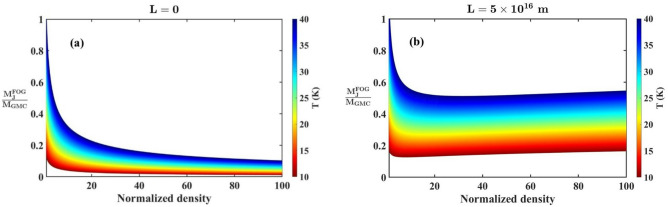
Profile of the normalized FOG-modified Jeans mass $\left( {M_{J}^{{FOG}}/{M_{GMC}}} \right)$ with variation in the normalized density for different *T*-values with (a) $L=0$ m and (b) $L=5 \times {10^{16}}$ m.

Again, Fig. 2 illustrates the variation of $M_{J}^{{FOG}}/{M_{GMC}}$, with the normalized material density for both the Newtonian case $\left( {L=0} \right)$ in Fig. 2a and the FOG case ($L=5 \times {10^{16}}$ m) in Fig. 2b. It is to be noted that the FOG predictions reduce to the Newtonian predictions in the limit $L \to 0$. This reduction is clearly explored in the supplementary information. The colour-gradient map represents different *T*-values. In Fig. 2a, corresponding to the Newtonian gravity scenario, the Jeans critical mass exhibits a relatively low temperature dependency. This is indicated by the limited vertical spread of the colour-gradient (*T*-values) across the background density range. Thus, it suggests that in the standard Newtonian gravity scenario, the *T*-variations have a relatively moderate influence on the critical mass required for triggering the gravitational collapse process, particularly in the high-density regime. On the contrary, Fig. 2b reveals a stronger *T*-variation dependency compared to the former case. In the FOG scenario, the colour gradients are more widely spaced at both the low and high-density regimes. These findings indicate that the *T*-variations have a significantly greater impact on the FOG-modified Jeans mass. This fundamental difference highlights the role of the FOG corrections in amplifying the regulatory role of the system temperature in determining the stability of various astrophysical structures.

The results reported in the graphical analyses above indicates the scale-dependent suppression of the self-gravitational Jeans instability. The emergence of a non-monotonic trend of $M_{J}^{{FOG}}$ with a higher *T*-value indicates a favourable collapse at an intermediate density. It also reveals that the influence of the cloud temperature on initiating the collapse process is significantly enhanced in the FOG scenario. These findings imply significant variation in the cloud fragmentation and the star-formation processes in astroclouds of similar densities but with different cloud temperatures. Consequently, this scale and temperature sensitive gravitational behaviours could provide a possible natural explanation for the star-formation activities across various molecular clouds^61,62^. Thus, the increased critical limit and the amplified thermal regulation introduced by the FOG theory extends the classical understanding of the collapse dynamics. It offers a coherent theoretical foundation for interpreting observational trends in structure-forming regions.

To further contextualize the theoretical predictions of the FOG theory, we compute and compare ${M_J}$ and $M_{J}^{{FOG}}$ for typical GMCs, diffuse molecular clouds and Bok globules. These astrophysical systems offer a wide range of physical properties, making them ideal candidates for investigating the impact of the FOG corrections in initiating the gravitational collapse processes across various scales. The key physical parameters.

of these astrophysical systems needed for computing the Jeans critical masses in both the frameworks are the cloud temperature $\left( T \right)$, hydrogen molecule number density $\left( {{n_{{H_2}}}} \right)$, and the cloud mass $\left( M \right)$.

The GMCs are massive and cold molecular cloud complexes, typically of $M\sim \left( {{{10}^4} - {{10}^6}} \right)~{M_ \odot }$ with ${n_{{H_2}}}$ ranging from $\left( {1 - 3} \right) \times {10^8}~{{\mathrm{m}}^{ - 3}}$^59,60^. The cloud temperature of these GMCs typically varies as $T\sim \left( {10 - 20} \right)$ K^31,59,60^. The diffuse molecular clouds are more thermally active compared to GMCs, with temperature varying as $T\sim \left( {15 - 50} \right)$ K^31^. The number density of these clouds is typically in the range given as ${n_{{H_2}}}\sim \left( {5 - 50} \right) \times {10^8}~{{\mathrm{m}}^{ - 3}}$ with cloud mass, $M\sim \left( {3 - 100} \right)~{M_ \odot }$^31^. These clouds are magnetized, turbulent and marginally self-gravitating, making them particularly interesting for testing various gravity models when these effects are taken into account. Again, the Bok globules are small, dense clouds typically found near the H II regions. These clouds are characterized by simple morphologies, relative isolation, and low cloud temperature of around $T\sim \left( {10 - 20} \right)$ K^30,42^. The cloud mass ranges from $M\sim \left( {2 - 50} \right)~{M_ \odot }$, with the particle number density as ${n_{{H_2}}}\sim 1 \times {10^{10}}~{{\mathrm{m}}^{ - 3}}$^30,31,42^. The masses of these Bok globules are typically comparable to their corresponding Jeans masses, making them highly sensitive to small variations in the critical threshold for gravitational collapse. Thus, the FOG-induced corrections made in the critical mass limits can lead to different predictions for the stability of these clouds.

**Table 1:** Jeans critical mass for different structure forming astroclouds in Newtonian and FOG framework.

S. No	Astroclouds	$T~\left( K \right)$	${n_{{H_2}}}{\mathrm{~}}\left( {{{\mathrm{m}}^{ - 3}}} \right)$	$M~\left[ {{M_ \odot }} \right]$	${M_J}~\left[ {{M_ \odot }} \right]$	$M_{J}^{{FOG}}~\left[ {{M_ \odot }} \right]$
1	Giant molecular cloud	15	$1 \times {10^8}$	${10^4} - {10^6}$	188.04	$606.64 - 1.27 \times {10^4}$
2	Diffuse molecular cloud	30	$5 \times {10^9}$	$3 - 100$	75.22	$77.37 - 130.86$
3	Bok globules	10	$1 \times {10^{10}}$	$2 - 50$	10.26	$11.37 - 28.46$

The Table 1 presents a comparison of the traditional Newtonian Jeans mass $\left( {{M_J}} \right)$ and the FOG-modified Jeans mass $\left( {M_{J}^{{FOG}}} \right)$ for the different astrocloudic structures discussed above. The results highlight the importance of the scale-dependent modifications in the FOG-framework influencing the gravitational fragmentation and astrostructure formation across various scales. For the case of the Bok globules and diffuse molecular clouds, the deviation of the Jeans critical masses in both the Newtonian and the FOG frameworks are relatively small, indicating moderate FOG corrections at smaller scales. However, in large systems, like GMCs, the deviation becomes highly pronounced, with $M_{J}^{{FOG}}=\left( {606.64 - 1.27 \times {{10}^4}} \right)~{M_ \odot }$, far exceeding the Newtonian prediction of ${M_J}=188.04{\mathrm{~}}{M_ \odot }$. This behaviour reflects a shift of the gravitational instability toward larger characteristic mass scales within the FOG framework, rather than an enhancement of collapse itself. Such a shift may influence the typical mass of initial fragments formed during the early stages of gravitational instability, within the assumptions of linear theory.

Various constraints on the *L*-value reported in the literature might predict non-significant deviations of the Jeans critical masses for these astroclouds. The tightest constraint is calculated from the periodic decay and periastron advance data of the *Hulse-Taylor pulsar*^46^. Its constraint is found to lie on the free-length parameter as $L \leqslant 4.42 \times {10^7}$ m. Such constraints probe local, strong-field astrophysical environments and essentially predict the Newtonian limits for initiating gravitational collapse processes at astrocloudic scales. In the present work, the analysis is intended to explore the phenomenological consequences of scale-dependent gravitational corrections in the weak-field regime, without asserting the observational viability of specific numerical values of the *L*-parameter. Accordingly, it is treated as an external parameter to examine the influence of higher-order gravity corrections on the instability threshold, rather than as a quantity fixed by local constraints. A comprehensive connection between the bounds derived from compact-object systems and those relevant for large-scale astrophysical environments would require a more complete theory and lies beyond the scope of this study.

**Table 2:** Jeans critical mass of different Bok globules in Newtonian and FOG formalism.

S. No	Bok globules	*T* $\left( K \right)$	${n_{{H_2}}}{\mathrm{~}}\left( {{{\mathrm{m}}^{ - 3}}} \right)$	$M~\left[ {{M_ \odot }} \right]$	${M_J}~\left[ {{M_ \odot }} \right]$	$M_{J}^{{FOG}}~\left[ {{M_ \odot }} \right]$
1	CB 87	11.4	$\left( {1.7 \pm 0.2} \right) \times {10^{10}}$	$2.73 \pm 0.24$	9.55	11.61
2	CB 110	21.8	$\left( {1.5 \pm 0.6} \right) \times {10^{11}}$	$7.21 \pm 1.64$	8.50	23.60
3	CB 131	25.1	$\left( {2.5 \pm 1.3} \right) \times {10^{11}}$	$7.83 \pm 2.35$	8.14	28.58
4	CB 134	13.2	$\left( {7.5 \pm 3.3} \right) \times {10^{11}}$	$1.91 \pm 0.52$	1.79	7.57
5	CB 161	12.5	$\left( {7.0 \pm 1.6} \right) \times {10^{10}}$	$2.79 \pm 0.72$	5.40	9.20
6	CB 184	15.5	$\left( {3.0 \pm 0.4} \right) \times {10^{10}}$	$4.70 \pm 1.76$	11.40	16.45
7	CB 188	19.0	$\left( {1.2 \pm 0.2} \right) \times {10^{11}}$	$7.19 \pm 2.28$	7.73	20.56
8	FeSt 1-457	10.9	$\left( {6.5 \pm 1.7} \right) \times {10^{11}}$	$1.12 \pm 0.23$	1.44	4.67
9	Lynds 495	12.6	$\left( {4.8 \pm 1.4} \right) \times {10^{10}}$	$2.95 \pm 0.77$	6.61	10.12
10	Lynds 498	11.0	$\left( {4.3 \pm 0.5} \right) \times {10^{10}}$	$1.42 \pm 0.16$	5.69	7.32
11	Coalsack	15.0	$\left( {5.4 \pm 1.4} \right) \times {10^{10}}$	$4.50$	8.09	13.94

Again, Table 2 presents a comparative analysis of ${M_J}$ and $M_{J}^{{FOG}}$ for several Bok globules reported in Ref.^42^. As expected, $M_{J}^{{FOG}}$ only increases marginally across the different Bok globules, thereby predicting a predominantly stable cloud configuration. This minimal difference in the critical mass arises due to the inherently small spatial and mass scales of the Bok globules. As the FOG theory introduces significantly higher-order corrections primarily in large-scale massive astrostructures, its stabilizing influence is effectively suppressed in smaller systems. This result is consistent with the scale-dependent nature of the FOG framework, where larger astroclouds like GMCs experience a substantial increase in their critical mass limit. However, the FOG theoretical predictions for the stability of the Bok globules stand in contrast with the observational data, showing active star-formation in many of these clouds^42^. This suggests that while the FOG theory offers a compelling framework for promoting large-scale structure formation, its applicability to small-scale systems remains limited. The collapse of Bok globules is likely governed by additional local mechanisms, such as external compression, turbulence, magnetic fields, and so forth. Thus, these findings highlight that, while the FOG corrections are negligible in small-scale systems, they emerge as a significant regulatory mechanism for the self-gravitational collapse in massive and large-scale astrophysical environments.

## FOG-modified dispersion results

In this section, we develop a comprehensive analytical framework to investigate the FOG-modified Jeans instability dynamics from a modal analysis perspective. We derive a modified dispersion relation capturing the higher-order curvature effects, extending the classical Newtonian analysis. In this context, Eq. (13) can be rewritten in the following form as

20 $$1+{L^2}{k^2} - \frac{{k_{J}^{2}}}{{{k^2}}}\frac{1}{{\sqrt {2\pi } }}\smallint \frac{{x{e^{ - {x^2}/2}}}}{{x - \beta }}dx=0,$$

where we have defined the variables

21 $$\beta =\frac{\omega }{{k\sigma }};{\mathrm{~}}x=\frac{v}{\sigma }.$$

In Eq. (20), the integration over *x* is carried out across the entire velocity domain, $x \in \left( { - \infty ,+\infty } \right)$, as prescribed by the Maxwell-Boltzmann distribution in the kinetic description. In the low-frequency regime $\left( {\beta \ll 1} \right)$, this integral can be expanded in powers of $\beta$, with the contribution from the pole $\left( {x=\beta } \right)$ evaluated using the Cauchy principal value. This calculation is directly related to the well-known plasma dispersion function, $Z\left( \beta \right)$^47^. The small-$\beta$ expansion yields a constant real term and an imaginary term linear in $\beta$. Thus, in the low-frequency limit, the integral in Eq. (20) can be recast as^35^

22 $$\frac{1}{{\sqrt {2\pi } }}\smallint \frac{{x{e^{ - {x^2}/2}}}}{{x - \beta }}dx \approx 1+i\sqrt {\frac{\pi }{2}} \beta .$$

Thus, Eq. (20) can be rewritten in the following form

23 $$1+{L^2}{k^2} - \frac{{k_{J}^{2}}}{{{k^2}}}~\left( {1+i\sqrt {\frac{\pi }{2}} \beta } \right)=0.$$

Now, in the context of investigating Jeans instability, the wave perturbations can grow due to self-gravitational attraction or dissipate due to the external thermal pressure. To capture these two behaviours, we consider a complex Jeans angular frequency. This treatment essentially captures the wave oscillation described by the real part $\left( {{\omega _r}} \right)$, and the change in the perturbation amplitude described by the imaginary part $\left( {{\omega _i}} \right)$. Thus, for investigating the perturbation dynamics we decompose $\omega$ as, $\omega ={\omega _r}+i{\omega _i}$; and set ${\omega _r}=0$ in Eq. (23), resulting in the following equation as

24 $${\omega _i}=k\sigma \sqrt {\frac{2}{\pi }} \left[ {1 - \left( {\frac{{{k^2}}}{{k_{J}^{2}}}+{L^2}\frac{{{k^4}}}{{k_{J}^{2}}}} \right)} \right].$$

Here, Eq. (24) represents the instability growth rate, which gives unstable modes when ${\omega _i}>0$^35,47^. In the unstable modes, the amplitude of the perturbation grows exponentially over time, leading to structure formation. The condition for the modes to be unstable can be determined from Eq. (24), and is cast as

25 $$\left( {\frac{{{k^2}}}{{k_{J}^{2}}}+{L^2}\frac{{{k^4}}}{{k_{J}^{2}}}} \right)<1.$$

The dimensionless form of Eq. (24) can be expressed by implementing a standard normalization scheme and is given by

26 $${\Omega _i}=\frac{{K{k_J}\sigma }}{{\sqrt {4\pi G{\rho _0}} }}\sqrt {\frac{2}{\pi }} \left[ {1 - \left( {{K^2}+{L^2}{K^4}k_{J}^{2}} \right)} \right].$$

Here, ${\Omega _i}={\omega _i}/{\omega _J}$ represents the normalized growth rate of perturbations, where ${\omega _J}=\sqrt {4\pi G{\rho _0}}$ denotes the Newtonian Jeans frequency. Similarly, $K=k/{k_J}$ denotes the normalized Jeans wavenumber.

**Fig. 3:**
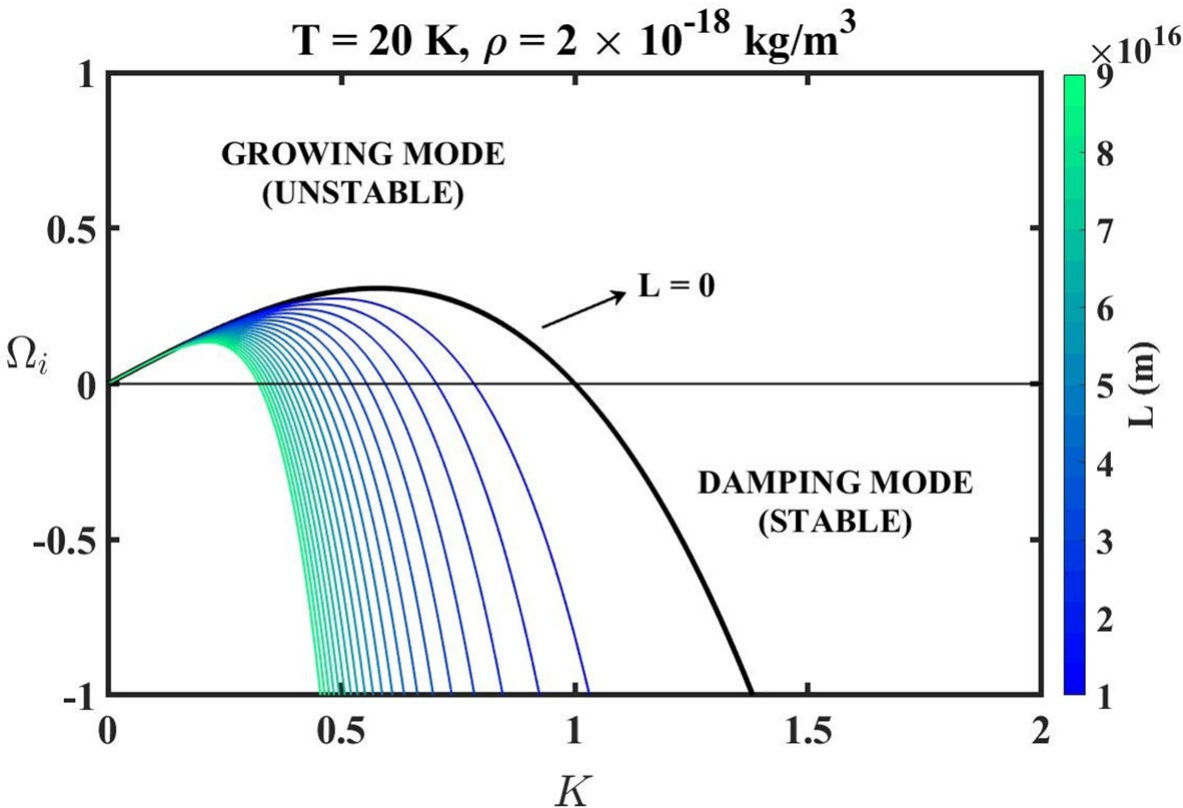
Profile of the normalized instability growth rate $\left( {{{\mathrm{\boldsymbol{\Omega}}}_i}} \right)$ with variation in the normalized wavenumber $\left( K \right)$ for the different indicated *L*-values.

Now, for $L=0$, Eq. (26) reduces to the corresponding expression obtained in the Newtonian gravity formalism^47^. From Eq. (26), we examine the growth rate of the perturbations by plotting the profile of the FOG-modified dispersion relation curve in Fig. 3. We use the parametric values of typical GMCs as the input parameters, $\left( {T=20~{\mathrm{K}},{\mathrm{\boldsymbol{\uprho}}}=2 \times {{10}^{ - 18}}~{\mathrm{kg~}}{{\mathrm{m}}^{ - 3}}} \right)$ for the graphical analysis of the modified dispersion relation.

The *L*-parametric variation of the instability growth rate is depicted in Fig. 3. Here, the unstable modes are indicated by a positive ${{\mathrm{\boldsymbol{\Omega}}}_i}$-value. For wavenumbers $K<{K_J}$, the perturbations are unstable and are termed as the growing modes. In these modes, the inward acting gravitational force dominates over the outward acting thermal pressure. These perturbative modes grow exponentially over time, leading to gravitational collapse and consequently forming astrostructures. Again, for wavenumbers $K>{K_J}$, the perturbations are stable $\left( {{{\mathrm{\boldsymbol{\Omega}}}_{\mathrm{i}}}<0} \right)$, and are thus termed as the damping modes. In this perturbation scale, the outward acting pressure dominates over the inward gravitational force, resulting in damped wave oscillations. Thus, these modes do not contribute to the structure formation processes. In the regime $K \to 0$, the wavelength of the perturbations effectively becomes infinite, contributing to the entire system being perturbed at a global scale. In such perturbations, the self-gravitational force is spread over an infinite region. Thus, the gravitational attraction is negligible as the mass is distributed over an arbitrary infinite volume. Consequently, no local density contrast develops in the system to trigger structure formation. This results in a zero-instability growth rate $\left( {{{\mathrm{\boldsymbol{\Omega}}}_i} \to 0} \right)$.

Additionally, we also observe that the peak instability growth rate diminishes, and the curves shift towards lower *K*-values. This indicates a stabilizing nature influenced by the *L*-parameter. Notably, the spacing between successive curves becomes increasingly compressed for higher *L*-values, suggesting a nonlinear but saturating effect of the FOG modifications. It implies that beyond a certain threshold, further increase in the *L*-value contributes minimally to the suppression of the gravitational instability. Consequently, it indicates the presence of an effective saturation scale of the *L*-parameter against the growth-narrowing-down effects. Physically, this reflects a weakening of the inward acting self-gravity at shorter scales, favouring the growth of only sufficiently long perturbations. The $L=0$ curve corresponds to the Newtonian gravity. It features the widest instability band and the highest growth rate, whereas increasing *L*-value steadily suppresses both.

**Fig. 4:**
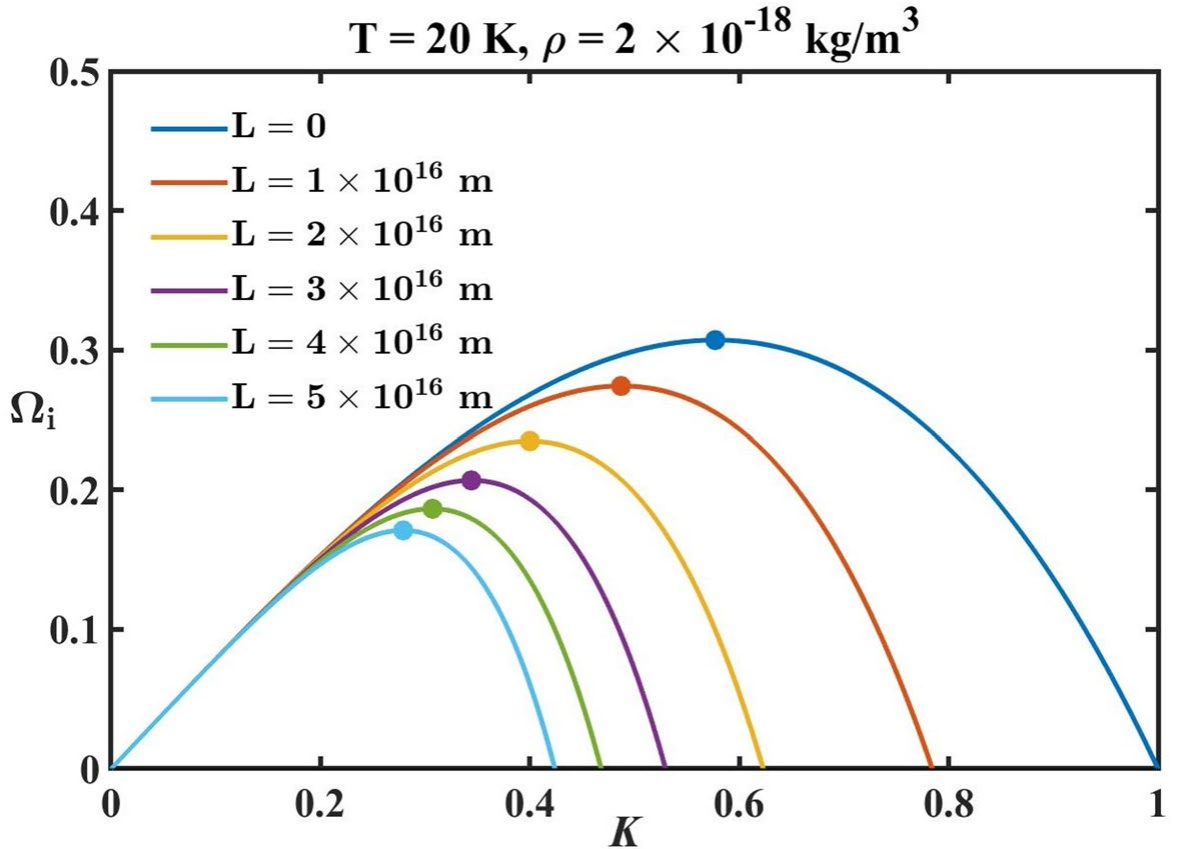
Profile of the normalized growth rate $\left( {{{\mathrm{\boldsymbol{\Omega}}}_i}} \right)$ with variation in the normalized wavenumber $\left( K \right)$ for different *L*-values in the unstable region $\left( {K<{K_J}} \right)$, with the normalized fastest growing mode indicated corresponding to each dispersion curve.

In Fig. 4, we plot the dispersion relation for the unstable modes for different indicated *L*-values. The maxima of each dispersion curve denote the normalized peak wavenumber $\left( {{K_m}} \right)$. It represents the wave perturbation with the highest growth rate in a system undergoing gravitational collapse. This mode corresponds to the most rapidly growing fluctuation leading to significant collapse and fragmentation. The mass associated with ${K_m}$ is termed the peak mass $\left( {{M_m}} \right)$. It represents the mass of the fragments or clumps that are most likely to be formed from the initial fragmentation process. From Fig. 4, we see that the ${K_m}$-value for each dispersion curve decreases with an increase in the *L*-value.

**Table 3:** Normalized fast-growing modal characteristics.

S. No	*L*-value	Peak growth rate $\left( {{\mathrm{\boldsymbol{\Omega}}}_{i}^{m}} \right)$	Peak wavenumber $\left( {{K_m}} \right)$	Peak mass $\left( {{M_m}/{M_{GMC}}} \right)$
1	0	$3.07 \times {10^{ - 1}}$	$5.77 \times {10^{ - 1}}$	$1.32 \times {10^{ - 1}}$
2	$1 \times {10^{16}}$ m	$2.74 \times {10^{ - 1}}$	$4.87 \times {10^{ - 1}}$	$2.19 \times {10^{ - 1}}$
3	$2 \times {10^{16}}$ m	$2.35 \times {10^{ - 1}}$	$4.00 \times {10^{ - 1}}$	$3.96 \times {10^{ - 1}}$
4	$3 \times {10^{16}}$ m	$2.07 \times {10^{ - 1}}$	$3.44 \times {10^{ - 1}}$	$6.22 \times {10^{ - 1}}$
5	$4 \times {10^{16}}$ m	$1.86 \times {10^{ - 1}}$	$3.07 \times {10^{ - 1}}$	$8.76 \times {10^{ - 1}}$
6	$5 \times {10^{16}}$ m	$1.71 \times {10^{ - 1}}$	$2.79 \times {10^{ - 1}}$	$1.17 \times {10^0}$

The modal characteristics of each dispersion curve plotted in Fig. 4 are shown in Table 3. It shows that the normalized peak growth mass $\left( {{M_m}/{M_{GMC}}} \right)$ increases with an increase in the *L*-value. This suggests that, although *L* acts as a stabilizing agent against small-scale collapse, it can potentially form larger initial fragments. This in turn, can produce more massive astrostructures in contrast to the Newtonian regime through hierarchical fragmentation processes. The peak growth modal analysis provides insights into the typical size and mass of the structures forming in astroclouds and stellar systems. It highlights the dominant scale of instability and may help in predicting the initial mass distribution of the resultant fragment. This initial mass can potentially dictate the type and nature of astrophysical structures formed.

Such scale-biased collapse in the FOG framework can modify the characteristic mass spectrum associated with prestellar core formation. The FOG theory effectively shifts the instability toward larger length and mass scales suggesting a corresponding modification of the Core Mass Function (CMF) toward higher mass scales. Consequently, such a shift in the CMF may, in turn, influence the statistical properties of the stellar Initial Mass Function (IMF), as the IMF inherit its shape from the distribution of the core masses formed during fragmentation. In the classical picture, the *Salpeter IMF* assumes a universal power-law behaviour of the form ${\mathrm{\boldsymbol{\upxi}}}\left( M \right) \propto {M^{ - 2.35}}$, with *M* being defined as the stellar mass^63^. This implicitly assumes a scale-invariant gravitational collapse process. In contrast, the presence of scale-dependent gravitational corrections in the FOG framework introduces a preferred mass scale, which may lead to departures from strict scale invariance and favour top-heavy mass distributions at the level of initial fragmentation.

It is worth noting that, other models, such as the turbulence-regulated Hennebelle-Chabrier (HC08) IMF, also predicts a top-heavy distribution by incorporating stochastic density fluctuations modulated by varying Mach numbers^64,65^. In contrast to the HC08 model, the FOG approach offers a deterministic, gravity-driven alternative explanation, where the scale-dependent gravity corrections become significant. While a detailed mapping between a modified CMF and the resulting stellar IMF requires nonlinear evolution and additional physical processes beyond the scope of this linear analysis, the presented results indicate that the FOG framework can influence the characteristic mass scales of initial cloud fragments.

Observational evidence of top-heavy stellar populations in certain astrophysical environments has been reported in multiple studies^66,67^. For instance, the high mass-to-light ratios observed in ultra-compact dwarf galaxies suggests a top-heavy IMF^66^. Additionally, the observations from dusty starburst galaxies across cosmic time indicates the dominance of massive stars in these galaxies, further supporting the notion of top-heavy IMF in such regions^67^. These environments are often characterized by high densities and large masses, where the FOG corrections are expected to be prominent. In this context, the increasing trend of ${M_m}$ with higher *L*-values suggests a possible connection between the FOG-modified Jeans instability mechanism and the emergence of top-heavy mass distributions. However, such comparisons should be regarded as qualitative, serving primarily to illustrate the potential astrophysical relevance of the present framework rather than as direct observational predictions.

The FOG-modified Poisson equation employed in the proposed model analysis is methodically derived within the non-relativistic weak-field regime of the FOG theory. It is applicable only in the limiting cases where the higher-order terms act as a perturbative correction over the leading Newtonian contribution. The astrophysical systems considered and analysed here, such as the molecular clouds, Bok globules, and so forth, lie well within this weak-gravity regime. Since the relative contribution of the fourth-order correction term grows with increasing *K*-values (corresponding to shorter spatial scales), the modified dispersion relation should be interpreted within the range of spatial scales where the weak-field approximation remains self-consistently valid. In particular, the chosen values of the free length *L*-parameter ensure that the higher-order contribution remains subdominant compared to the Newtonian term within the Jeans-unstable regime under consideration. In the present analysis, a particular emphasis is placed on the Jeans-unstable region $\left( {K<1} \right)$, illustratively (Fig. 4), where the material density perturbations grow. In this regime, the perturbative description remains valid. The broader range of the normalized wavenumber, as displayed in Fig. 3, is included to illustrate the overall instability growth trends. However, the physical interpretation of the Jeans instability is restricted within the scales consistent with the effective weak-field framework only.

Building on these results, illustrative astrophysical contexts relevant to the FOG-modified Jeans instability are discussed in the supplementary information.

## Conclusions

This semi-analytic investigation offers a comprehensive study of the gravitational instability excitable in astrocosmic media within the FOG framework. It focuses mainly on the scale-dependent effects of the FOG theory on the non-local self-gravitational collapse dynamics in diverse astrophysical scenarios. The FOG-modified Jeans critical mass limit is systematically derived and illustratively discussed. The scale-dependent FOG framework introduces modifications to the Jeans mass, which depends on the initial mass of the system. A key result emerging from our analysis is the non-monotonic behaviour of the FOG-modified critical Jeans mass, $M_{J}^{{FOG}}$, with respect to the background matter density for high *L*-values. Consequently, the newly modified $M_{J}^{{FOG}}$ reaches a minimum at an intermediate density value. Beyond this limit, $M_{J}^{{FOG}}$ continues to increase again. It implies a canonical self-gravitational collapse within a restricted density regime of astronomical significance. This is qualitatively consistent with trends discussed in diverse observational studies previously reported elsewhere^66,67^.

A new dispersion relation is also derived, considering the FOG framework for a modal analysis of the gravitational instability in both self-gravitating astroclouds and stellar systems. In this gravity framework, larger astrophysical systems, such as GMCs experiences a greater deviation from the Newtonian Jeans mass, significantly influencing the cloud stability. It potentially favours the formation of larger initial fragments and fewer astrostructures through hierarchical fragmentation processes. Consequently, the FOG theory may also find its non-trivial applicability and relevance in the study of the observed delayed collapse of GMCs, a phenomenon not entirely accounted for in the classical Newtonian gravity theory^68^.

Although the present study is restricted to linear perturbations, the scale-dependent nature of the FOG-modified Jeans criterion suggests possible implications for the formation of large-scale structures such as galactic superclusters and cosmic filaments, which are some of the largest observed structures in the Universe^69,70^. Comparisons with the observed large-scale structures and massive stellar environments should be regarded as qualitative at this stage, serving primarily to illustrate the potential astrophysical relevance of the framework rather than as direct observational predictions. A comprehensive assessment of such connections would require nonlinear modelling, numerical simulations, and the inclusion of additional physical processes.

More broadly, the formalism developed here provides a general framework for analysing Jeans instability within effective higher-derivative theories of gravity using kinetic theory. As such, it may serve as a benchmark for future investigations aimed at connecting modified gravity models with kinetic descriptions of gravitational instability in astrophysical and cosmological settings. Further extensions incorporating nonlinear dynamics and observational constraints will be essential for assessing the full implications of the FOG theory on the structure formation mechanism.

## Supplementary Information

Below is the link to the electronic supplementary material.

Supplementary Material 1

## Data Availability

All data generated or analysed during this study are included in this published article [and its supplementary information files].
