# Supplementary material for: Kinetic Jeans instability in FOG framework
Source: Sci Rep. 2026 Mar 18;16:14103. doi: 10.1038/s41598-026-44639-6 (PMC13136324; doi:10.1038/s41598-026-44639-6)
Supplement: Supplementary file 1 — Supplementary Material 1 [file 41598_2026_44639_MOESM1_ESM.pdf]

SUPPLEMENTARY INFORMATION-1:
FOG-MODIFIED JEANS WAVENUMBER

In the traditional Jeans instability analysis founded on Newtonian gravity, there is only one critical Jeans limit for unstable modes in the angular wavenumber space. In contrast, for a more general FOG scenario, introducing higher-order corrections formally yields two mathematical solutions for the critical wavenumber at which the instability gets triggered. Thus, the two distinct k_J-solutions obtained from Eq. (16) in the main text can respectively be cast in a standard symbolism as

$$k_{J_+}^{FOG} = \left(\frac{-\sigma^2+\sqrt{\sigma^4+16L^2\sigma^2\pi G\rho_0}}{2L^2\sigma^2}\right)^{1/2}, \tag{S1.1}$$

$$k_{J_-}^{FOG} = \left(\frac{-\sigma^2-\sqrt{\sigma^4+16L^2\sigma^2\pi G\rho_0}}{2L^2\sigma^2}\right)^{1/2}. \tag{S1.2}$$

Although there exist two roots, only the positive real solution $\left(k_{J_+}^{FOG}\right)$ has a physical significance in our analysis. The second one $\left(k_{J_-}^{FOG}\right)$ is purely an imaginary and hence, fails to signify a valid instability onset threshold. This feature reflects that, although higher-order gravity theories, like the FOG model, can yield extra analytic branches, not all of these solutions are physically realizable.

In the following, we provide a mathematical analysis showing that, only $k_{J_+}^{FOG}$ and the associated mass limit, $M_{J_+}^{FOG}$, reduces to the standard Newtonian results in the asymptotic limit $L \to 0$. First, we analyse the positive real solution, $k_{J_+}^{FOG}$, defined by Eq. (S1.1). The numerator and the denominator inside the square root of Eq. (S1.1), as explicit functions of L, can respectively be cast as

$$f(L) = -\sigma^2 + \sqrt{\sigma^4 + 16L^2\sigma^2\pi G\rho_0}, \tag{S1.3}$$

$$g(L) = 2L^2\sigma^2. \tag{S1.4}$$

In the above limit $(L \to 0)$, both $f(L)$ and $g(L)$ approach to zero, leading to an intermediate form of the $0/0$-type [1]. To resolve the intermediate form, we apply L'Hôpital's rule (Bernoulli's rule) to find its limiting value. The first-order derivatives of Eqs. (S1.3)-(S1.4) are respectively cast as

$$f'(L) = \frac{16L\sigma^2\pi G\rho_0}{\sqrt{\sigma^4+16L^2\sigma^2\pi G\rho_0}}, \tag{S1.5}$$

$$g'(L) = 4L\sigma^2. \tag{S1.6}$$

Applying the L'Hôpital rule in Eqs. (S1.5)-(S1.6), one evaluates the limiting value as

$$\lim_{L\to 0}\left[\left(k_{J_+}^{FOG}\right)^2\right] = \lim_{L\to 0}\frac{f'(L)}{g'(L)} = \lim_{L\to 0}\frac{4\pi G\rho_0}{\sqrt{\sigma^4+16L^2\sigma^2\pi G\rho_0}}. \tag{S1.7}$$

Since, in the Newtonian limit $(L \to 0)$, the denominator in Eq. (S1.7) reduces to σ^2. Thus, we obtain the standard Newtonian Jeans wavenumber from Eq. (S1.7) as

$$\lim_{L\to 0} k_{J_+}^{FOG} = k_J = \frac{\sqrt{4\pi G\rho_0}}{\sigma}. \tag{S1.8}$$

This result confirms that $k_{J_+}^{FOG}$represents a physically meaningful generalization of the Newtonian Jeans wavenumber in the FOG formalism in the same kinetic picture. As a consequence, we can also infer that M_{J+}^{FOG} reduces to the Newtonian Jeans mass $\left(M_J\right)$ in the regime of $L \to 0$.

We further investigate the second solution $\left(k_{J_-}^{FOG}\right)$ as $L \to 0$, defined by Eq. (S1.2). In this limiting case, the numerator in Eq. (S1.2) strictly takes a negative value. Thus, for all values of L, the term inside the square root of Eq. (S1.2) remains negative. Since the Jeans wavenumber must be a real quantity for physical viability, the second solution ($k_{J_-}^{FOG}$) is not physically defined. Thus, this indicates that the Newtonian limit for this wavenumber does not exist. Therefore, from our mathematical analysis, we conclude that only $k_{J_+}^{FOG}$ smoothly reduces to the Newtonian Jeans wavenumber in the limit $L \to 0$. Consequently, the corresponding Jeans mass, $M_{J_+}^{FOG}$, can be signified as the generalization of the critical Jeans mass as the onset Jeans instability threshold in the traditional Newtonian picture.

SUPPLEMENTARY INFORMATION-2:
FOG-MODIFIED JEANS MASS

The non-monotonic density dependency of $M_J^{FOG} = f(\rho_0, L)$ is seen by analysing the derived FOG-modified Jeans mass with its asymptotic behaviours in both the low- and high-density regimes as

$$M_J^{FOG} = \left(\frac{4\rho_0\pi^4}{3}\right)\left(\frac{2L^2\sigma^2}{-\sigma^2+\sqrt{\sigma^4+16L^2\sigma^2\pi G\rho_0}}\right)^{3/2}. \tag{S2.1}$$

To understand the non-monotonic density dependence, we define an auxiliary function as

$$g(\rho_0, L) = \frac{2L^2\sigma^2}{-\sigma^2+\sqrt{\sigma^4+16L^2\sigma^2\pi G\rho_0}}. \tag{S2.2}$$

Thus, in the light of Eq. (S2.2), M_J^{FOG} defined by Eq. (S2.1) can be recast as

$$M_J^{FOG} = \left(\frac{4\rho_0\pi^4}{3}\right)[g(\rho_0)]^{3/2}. \tag{S2.3}$$

Now, at the low-density regime ($\rho_0/\rho_{\mathrm{GMC}} \ll 1$), the term $\sqrt{\sigma^4 + 16L^2\sigma^2\pi G\rho_0}$ in Eq. (S2.2) can be expanded using a first-order Taylor series. Firstly, we factor out σ^4 from the said term

$$\sqrt{\sigma^4 + 16L^2\sigma^2\pi G\rho_0} = \sigma^2\sqrt{1 + \frac{16\pi G\rho_0 L^2}{\sigma^2}}. \tag{S2.4}$$

In this limit, $16\pi G\rho_0 L^2/\sigma^2 \ll 1$, this allows the application of the binomial approximation $\left(\sqrt{1+\epsilon} \approx 1 + \epsilon/2\right)$. Here, we identify $\epsilon = 16\pi G\rho_0 L^2/\sigma^2$. Thus, expanding Eq. (S2.4) using first-order Taylor series, we obtain the approximate expression as

$$\sqrt{\sigma^4 + 16L^2\sigma^2\pi G\rho_0} \simeq \sigma^2 + 8\pi G\rho_0 L^2. \tag{S2.5}$$

Substituting the result of Eq. (S2.5) into $g(\rho_0, L)$ defined by Eq. (S2.2) we obtain,

$$g(\rho_0) = \frac{\sigma^2}{4\pi G\rho_0}. \tag{S2.6}$$

Thus, in this low-density regime, Eq. (S2.3) reduces to

$$M_J^{FOG} = \left(\frac{4\rho_0\pi^4}{3}\right)\left(\frac{\sigma^2}{4\pi G\rho_0}\right)^{3/2}, \tag{S2.7}$$

$$M_J^{FOG} \propto \rho_0^{-1/2}. \tag{S2.8}$$

Here, we see that in the low-density regime, M_J^{FOG} recovers the classical Newtonian Jeans mass density dependency. Thus, the system behaves as predicted by the Newtonian gravity theory. The Jeans critical mass decreases monotonically with increasing material density.

Again, at the high-density regime ($\rho_0/\rho_{\mathrm{GMC}} \gg 1$), in contrast to the former case, the denominator of the Eq. (S2.2) becomes dominated by the ρ_0-dependent component and thus, the expression simplifies to

$$g(\rho_0) = \frac{2L}{\sqrt{16\pi G\rho_0}}. \tag{S2.9}$$

Therefore, in the high-density regime, Eq. (S2.3) reduces to

$$M_J^{FOG} = \left(\frac{4\rho_0\pi^4}{3}\right)\left(\frac{2L}{\sqrt{16\pi G\rho_0}}\right)^{3/2}, \tag{S2.10}$$

$$M_J^{FOG} \propto L^{3/2}\rho_0^{1/4}. \tag{S2.11}$$

This result indicates a reversal in the expected trend of M_J^{FOG} with increasing material density. The scaling relation (Eq. (S2.11)), reveals that the critical Jeans mass increases with increase in the material density of the system. Also, it is noted that, the increase in the M_J^{FOG}-value with increasing density is scaled and strongly influenced by the L-parameter. Such behaviour is inherently non-Newtonian and arises due to the implementation of the higher-order curvature corrections introduced by the FOG theory.

In conclusion, the asymptotic behaviour of M_J^{FOG} in both the low- and high-density scenarios reveal a distinctive departure from the classical Newtonian gravitational collapse predictions. In the low-density regime, M_J^{FOG} follows the classical Newtonian scaling $\left(M_J^{FOG} \propto \rho_0^{-1/2}\right)$, whereas, in the high-density regime, M_J^{FOG} exhibits a modified scaling behaviour $\left(M_J^{FOG} \propto L^{3/2}\rho_0^{1/4}\right)$, where the impact of the L-parameter becomes increasingly pronounced. This transition in the scaling behaviour gives rise to a non-monotonic dependence of M_J^{FOG} on the material density.

SUPPLEMENTARY INFORMATION-3: ASTRONOMICAL APPLICATIONS IN FOG

Here we outline some possible astrophysical contexts in which the derived FOG-modified Jeans instability criterion may be relevant. These considerations are intended to be illustrative rather than predictive, as the present work focuses on linear instability analysis and does not incorporate nonlinear evolution, feedback, or additional microphysical processes. The inclusion of higher-order gravity corrections modifies both the critical limits for gravitational collapse and the range of excited unstable modes. Nevertheless, the modified critical limits obtained here suggest several avenues where higher-derivative gravitational effects could influence various astrophysical environments.

3.1. Bok globules probing L-parameter

Bok globules are small, dense, and relatively isolated molecular clouds that are traditionally treated as testing grounds for the Jeans instability analysis. Observations reveal active star formation in many such globules, whereas Newtonian gravity often predicts marginal stability [2,3]. This discrepancy makes them an appealing laboratory for testing modifications to the collapse criterion.

In the FOG framework, the higher-order correction introduces a stabilizing contribution to the gravitational collapse. As a result, several Bok globules that are observed to undergo collapse would be classified as stable within the FOG framework, as already discussed in the section titled "FOG-modified Jeans mass". It indicates that the FOG formalism does not enhance collapse on small scales but instead may provide a qualitative upper bound on the L-parameter. The observed collapse of the recorded Bok globules can potentially place an upper bound on the admissible values of the L-parameter. If the L-value is too small, the Newtonian limit is recovered, and if the L-value is too large, the collapse process is suppressed contradicting the observations. We note that other astrophysical systems have also been employed to constrain the L-value [4,5]. Here we emphasize mainly on the fact that the Bok globules can serve as an additional, independent probe for the purpose of constraining the L-value.

It is also important to note that the Bok globule dynamics are strongly influenced by additional local processes including turbulence, magnetic fields, and external compression. These processes are not included in our purely gravitational treatment. These factors may provide an easier pathway for trigger the collapse processes leading to structure formation.

3.2. Massive star formation and compact objects

In dense molecular cloud regions, the modified collapse threshold alters the typical mass of the gravitationally bound fragments. A shift toward larger fragment masses may influence the relative abundance of massive stars. These massive stars can evolve into neutron stars, black holes, and may serve as progenitors to gamma-ray bursts (GRBs) [6]. Although this work does not model the stellar populations explicitly, it is possible to compare the CMFs in extreme star-forming environments with predictions based on the FOG framework. Such a comparison would remain qualitative and could serve as indirect probes of the FOG effects.

3.3. Fragmentation in accretion disks

In self-gravitating disks surrounding black holes and neutron stars, the instability thresholds set the conditions and locations for fragmentation [7]. The scale-dependent modifications from the FOG theory shifts the fragmentation scale, potentially affecting the mass and frequency of infall events onto the central gravitating objects. Such changes could, in principle, influence accretion-powered high-energy

emissions, including X-ray variability [8] and jet-launching conditions [9]. High-resolution imaging of AGN disks or circumstellar disks in X-ray binaries could be used to identify such shifts.

3.4. High-redshift quasars and black hole seeds

In the high-redshift Universe ($z \gtrsim 6$), the modified collapse scale may promote the formation of more massive early gas clouds, thereby facilitating the rapid assembly of black hole (BH) seeds. The origin of such seeds and their ability to attain large masses on short cosmic timescales remain key open problems in astrophysics [10]. These early black hole seeds are capable of powering luminous quasars at $z \gtrsim 6$, which are prominent UV-X-ray emitters. The observed number counts and spatial distribution of these objects can serve as statistical probes for evaluating the consistency of the FOG-modified collapse criterion with the massive black hole population in the early Universe.

3.5. Astrocosmic observational pathways

The dispersion relation derived in this work (Eq. (26)) provides a theoretical basis for exploring the effects of the FOG-modified collapse scales in influencing various astrophysical systems relevant to high-energy phenomena. While this study does not attempt a full numerical or observational verification, the scale-dependent nature of the instability threshold established in this work suggests several realistic avenues for future testing. Table. 3.1. summarizes these suggested applications, indicating the type of astrophysical system, the possible effects implied by the present FOG framework, the associated observables, and the relevant instruments most suited to acquiring the necessary data.

Table 3.1: Observational pathways for testing FOG-modified Jeans instability

S. No	Astrosystem	FOG impact	Key observable	Astronomical mission
1	Dense star-forming regions	Shift in characteristic core mass; altered massive star fraction	CMF and IMF slope	ALMA [11], VLA [12], JWST NIRCam/MIRI [13]
2	Compact-object populations	Changes in remnant core masses; variation in merger rates	GW source mass distributions	LIGO–Virgo [14], KAGRA [15]
3	Accretion disks (AGN / XRBs)	Change in fragmentation radius; modified feeding patterns	Clump locations in disks; X-ray variability patterns	ALMA [11], JWST [13], Chandra [16], XMM-Newton [17], NICER [18]
4	High-redshift quasars	Early BH seed formation; variation in quasar clustering	Quasar counts and spatial correlation at $z \gtrsim 6$	SDSS [19], DESI [20], Euclid [21,22], eROSITA [23]
5	Early large-scale structures	Influence on small-scale collapse; change in power spectra	Power spectrum features; Lyman-α forest; etc.	DESI [20], Subaru PFS [24], SKA [25]
6	Molecular cloud filaments	Modification in fragmentation length; core-filament spacing	Core separation statistics; filament mass-length map	ALMA [11], JWST [13]
7	Dwarf galaxies and small halos	Suppression or enhancement of gravitational collapse	Halo mass function; satellite galaxy counts	Euclid [21,22], SKA [25]

References

1. Simmons, G. F. *Calculus with Analytic Geometry*. (McGraw-Hill, 1996).
2. Ourabah, K. Jeans analysis in fractional gravity. *The European Physical Journal C* **84**, 1047 (2024).
3. Kandori, R. *et al.* Near-Infrared Imaging Survey of Bok Globules: Density Structure. *Astron. J.* **130**, 2166–2184 (2005).
4. Banerjee, S., Bera, S., Banerjee, S. & Singh, T. P. Constraints on fourth order gravity from binary pulsars and gravitational waves. *Physical Review D* **96**, (2017).
5. Banerjee, S., Jayswal, N. & Singh, T. P. Cosmic acceleration in a model of fourth order gravity. *Physical Review D - Particles, Fields, Gravitation and Cosmology* **92**, (2015).
6. Woosley, S. E. & Bloom, J. S. The Supernova–Gamma-Ray Burst Connection. *Annu. Rev. Astron. Astrophys.* **44**, 507–556 (2006).
7. Goodman, J. Self-gravity and quasi-stellar object discs. *Mon. Not. R. Astron. Soc.* **339**, 937–948 (2003).
8. Ishibashi, W. & Courvoisier, T. J.-L. X-ray variability time scales in active galactic nuclei. *Astron. Astrophys.* **504**, 61–66 (2009).
9. Punsly, B. First image of a jet launching from a black hole accretion system: Kinematics. *Astron. Astrophys.* **685**, L3 (2024).
10. Volonteri, M. Formation of supermassive black holes. *The Astronomy and Astrophysics Review* **18**, 279–315 (2010).
11. Partnership, A. *et al.* THE 2014 ALMA LONG BASELINE CAMPAIGN: AN OVERVIEW. *Astrophys. J.* **808**, L1 (2015).
12. Perley, R. A., Chandler, C. J., Butler, B. J. & Wrobel, J. M. THE EXPANDED VERY LARGE ARRAY: A NEW TELESCOPE FOR NEW SCIENCE. *Astrophys. J.* **739**, L1 (2011).
13. Rieke, M. J., Kelly, D. & Horner, S. Overview of James Webb Space Telescope and NIRCam's Role. in (eds. Heaney, J. B. & Burriesci, L. G.) 590401 (2005). doi:10.1117/12.615554.
14. Abbott, B. P. *et al.* Observation of Gravitational Waves from a Binary Black Hole Merger. *Phys. Rev. Lett.* **116**, 061102 (2016).
15. Akutsu, T. *et al.* Overview of KAGRA: Detector design and construction history. *Progress of Theoretical and Experimental Physics* **2021**, (2021).
16. Weisskopf, M. C. *et al.* An Overview of the Performance and Scientific Results from the *Chandra X-Ray Observatory*. *Publications of the Astronomical Society of the Pacific* **114**, 1–24 (2002).
17. Jansen, F. *et al.* XMM-Newton observatory. *Astron. Astrophys.* **365**, L1–L6 (2001).
18. Gendreau, K. C. *et al.* The Neutron star Interior Composition Explorer (NICER): design and development. in (eds. den Herder, J.-W. A., Takahashi, T. & Bautz, M.) 99051H (2016). doi:10.1117/12.2231304.

19. Tegmark, M. *et al.* Cosmological parameters from SDSS and WMAP. *Physical Review D* **69**, 103501 (2004).

20. DESI Collaboration *et al.* Overview of the Instrumentation for the Dark Energy Spectroscopic Instrument. *Astron. J.* **164**, 207 (2022).

21. Hunt, L. K. *et al. Euclid*: Early Release Observations – Deep anatomy of nearby galaxies. *Astron. Astrophys.* **697**, A9 (2025).

22. Cuillandre, J.-C. *et al. Euclid*: Early Release Observations – Programme overview and pipeline for compact- and diffuse-emission photometry. *Astron. Astrophys.* **697**, A6 (2025).

23. Krippendorf, S. *et al.* The eROSITA Final Equatorial-Depth Survey (eFEDS): A machine learning approach to inferring galaxy cluster masses from eROSITA X-ray images. *Astron. Astrophys.* **682**, A132 (2024).

24. Takada, M. *et al.* Extragalactic science, cosmology, and Galactic archaeology with the Subaru Prime Focus Spectrograph. *Publications of the Astronomical Society of Japan* **66**, (2014).

25. Dewdney, P. E., Hall, P. J., Schilizzi, R. T. & Lazio, T. J. L. W. The Square Kilometre Array. *Proceedings of the IEEE* **97**, 1482–1496 (2009).
